# Insurance Coverage Transitions After Disenrollment From Medicaid in Minnesota

**DOI:** 10.1001/jamanetworkopen.2023.9379

**Published:** 2023-04-21

**Authors:** Chris Frenier, Adrianna McIntyre

**Affiliations:** 1 Department of Health Policy and Management, Yale School of Public Health, New Haven, Connecticut; 2 Department of Health Policy and Management, Harvard T. H. Chan School of Public Health, Boston, Massachusetts

## Abstract

This cohort study examines insurance transitions after Medicaid disenrollment for Minnesota residents aged 64 years or younger.

## Introduction

Little is known about where, or how quickly, people gain new health insurance after exiting Medicaid.^1,2^ Understanding these dynamics may be particularly important as Medicaid continuous coverage, a pandemic-relief policy, is discontinued and states resume eligibility redeterminations. Estimates suggest 18 million people will exit Medicaid coverage during this process.^3^ This study examines insurance transitions after Medicaid disenrollment in a Medicaid expansion state with relatively generous continuous coverage and eligibility policies.^4^

## Methods

This cohort study used the monthly enrollment records of the Minnesota All Payer Claims Database (MN APCD) for Minnesota residents aged 64 years or younger (aged 0-64 years) with Medicaid enrollment spells ending between January 1, 2018, and February 28, 2019; data were accessed through partnership with the Minnesota Department of Health. The MN APCD includes commercial insurance, Medicaid (fee for service and managed care), and Medicare (fee for service and Medicare Advantage) records. The MN APCD enrollment data do not include information about enrollees’ race or ethnicity. We recorded individuals’ coverage type 6 and 12 months after Medicaid disenrollment. We coded enrollees as lost to follow-up if they were never observed in coverage between disenrollment and the latest month of available data (September 2021). Reasons for loss to follow-up include persistent uninsurance after leaving Medicaid, death, relocation outside Minnesota, or enrollment in nonreporting plan types (including some self-funded commercial plans).^5^ See the eMethods in Supplement 1 for more details on data limitations and sample construction. The Yale University Institutional Review Board waived approval for this study because it was deemed not human participant research; therefore, patient consent was not required. This study followed the STROBE reporting guideline.

## Results

The final sample included 346 734 Medicaid disenrollments (Table). Enrollees had a mean (SD) age of 26.4 (17.2) years at disenrollment, with 182 725 (52.7%) female and 164 009 (47.3%) male enrollees. Most had a Medicaid managed care plan during their last month of coverage; 20.5% of adults were enrolled in the state’s basic health plan (MinnesotaCare). We were able to match 204 345 cases (58.9%) to an enrollment record between Medicaid termination and the latest month of enrollment data. We coded the remaining 142 389 observations (41.1%) as lost to follow-up, noting that these individuals may have been uninsured or enrolled in nonreporting plans.

**Table.** Demographic Characteristics of Enrollees Exiting Medicaid Coverage, January 1, 2018, to February 28, 2019^a^

Characteristic	All (N = 346 734)	Children (n = 115 055)	Adults (n = 231 679)
Age, mean (SD), y^b^	26.4 (17.2)	7.9 (5.1)	35.6 (13.2)
Sex			
Female	182 725 (52.7)	55 935 (48.6)	126 790 (54.7)
Male	164 009 (47.3)	59 120 (51.4)	104 889 (45.3)
Lost to follow-up^c^	142 389 (41.1)	51 230 (44.5)	91 159 (39.3)
Coverage type when disenrolled			
Fee-for-service Medical Assistance^d^	55 965 (16.1)	20 550 (17.9)	35 415 (15.3)
Prepaid Medical Assistance^e^	237 835 (68.6)	93 546 (81.3)	144 289 (62.3)
Special Needs Basic Care^f^	4510 (1.3)	NA	4510 (1.3)
MinnesotaCare^g^	48 424 (14.0)	959 (0.8)	47 465 (20.5)

Abbreviation: NA, not applicable.

^a^ Data are the authors’ analysis of the Minnesota All Payer Claims Database and are presented as number (percentage) unless otherwise indicated.

^b^ Enrollees were classified as adults (aged 19-64 years) or children (aged 0-18 years) based on their age during the last month of coverage.

^c^ Lost to follow-up includes enrollees who were not observed in coverage at any point between disenrollment and September 2021. These data could be attributable to long-term uninsurance, relocation from Minnesota, death, data linkage error, or enrollment in a nonreporting plan type.

^d^ Fee-for-service Medical Assistance is the combined Medicaid/Children’s Health Insurance Program administered by Minnesota Department of Human Services.

^e^ Prepaid Medical Assistance is the Medicaid/Children’s Health Insurance Program managed care program for enrollees in risk-bearing managed care organizations.

^f^ Special Needs Basic Care is a Medicaid managed care program for people with disabilities.

^g^ MinnesotaCare is the state’s Patient Protection and Affordable Care Act Basic Health Program.

The Figure shows insurance coverage among 140 520 adult (Figure, A) and 63 825 child (Figure, B) observations that were not lost to follow-up at 6 and 12 months after Medicaid disenrollment. Six months after leaving Medicaid, 17.6% of children had enrolled in commercial group coverage, and 2.2% of children had enrolled in individual coverage. Among children, 48.7% did not have identifiable MN APCD coverage at 6 months, decreasing to 29.8% after 12 months, largely because of Medicaid reenrollment; 51.2% of children who left the program were reenrolled 1 year later.

**Figure.**
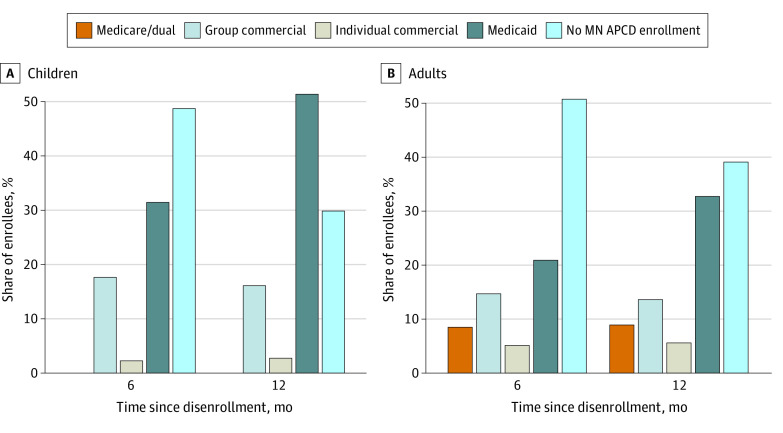
Insurance Coverage Following Disenrollment From Medicaid, January 1, 2018, to February 28, 2019 MN APCD indicates Minnesota All Payer Claims Database.

Among adults, 50.1% did not have identifiable coverage 6 months after Medicaid exit, decreasing to 39.1% at 12 months. The share of adults covered by commercial coverage after 12 months was 13.6% for group plans and 5.9% for individual plans. The share who had returned to Medicaid at 12 months was 32.8%.

## Discussion

This study found that approximately half of people disenrolling from Medicaid in Minnesota had no observable coverage 6 months later, and a substantial share returned to Medicaid within 1 year. Many enrollees failed to seamlessly transition to new coverage, and a meaningful share of disenrollment may have been among enrollees who were eligible for Medicaid or experienced short-term changes in eligibility that did not persist for a full year. These transitions incur administrative costs for states and can disrupt access to care.^6^

A limitation of our study is the inability to observe certain coverage outcomes. The MN APCD is estimated to include 40% of commercial enrollment during the study period, so some of those categorized as having no MN APCD enrollment may have been enrolled in nonreporting commercial plans, resulting in underestimation of true group commercial enrollment.

## Notes

Supplement 1. **eMethods.** Sample Construction and the Minnesota All Payer Claims Database

Supplement 2.
Data Sharing Statement
