# Supplementary material for: Insurance Coverage Transitions After Disenrollment From Medicaid in Minnesota
Source: JAMA Netw Open. 2023 Apr 21;6(4):e239379. doi: 10.1001/jamanetworkopen.2023.9379 (PMC10122164; doi:10.1001/jamanetworkopen.2023.9379)
Supplement: Supplement 1. — eMethods. Sample Construction and the Minnesota All Payer Claims Database [file jamanetwopen-e239379-s001.pdf]

Supplementary Online Content

Frenier C, McIntyre A. Insurance coverage transitions after disenrollment from Medicaid in Minnesota. *JAMA Netw Open*. 2023;6(4):e239379. doi:10.1001/jamanetworkopen.2023.9379

eMethods. Sample Construction and the Minnesota All Payer Claims Database

This supplementary material has been provided by the authors to give readers additional information about their work.

© 2023 Frenier C et al. *JAMA Network Open.*

eMethods. Sample Construction and the Minnesota All Payer Claims Database

The Minnesota All Payer Claims Database (MN APCD) is administered by the Health Economics Program at the Minnesota Department of Health (MDH). Public and private payers are statutorily required to submit de-identified claims and enrollment data for Minnesota residents to the MN APCD data vendor, Onpoint Health Data, on a regular basis. Onpoint uses a proprietary algorithm to link enrollment and claims data for the same individual across multiple submitters to generate a consolidated enrollment table at the person-month level that reports each individual's primary health and pharmacy coverage and any Medicaid, Medicare, and/or Commercial coverage they had in each month. We used this table to construct the analytic sample.

First, we identified non-elderly Minnesotans (under age 65) who exited Medicaid between January 2018 and February 2019. The study period was chosen so that our observation window did not include any months when COVID-19 related continuous coverage provisions were in effect. We classified any Minnesota Health Care Program enrollment as "Medicaid," which included Medicaid, State Children's Health Insurance Program, and MinnesotaCare (Basic Health Program). We defined exit from Medicaid as a month where an individual's primary health plan was Medicaid that was followed by at least two consecutive months with no Medicaid enrollment. We allowed for two-month interruptions in Medicaid coverage to account for data submission/linkage errors or short-term disenrollment that may have been subject to retroactive eligibility. We restricted the sample to individuals who were enrolled in Medicaid as their primary coverage for at least three months prior to exit.

We identified post-disenrollment coverage using enrollment records from the twelve months after the disenrollment date. In each month, we classified coverage into Medicare, Medicaid, Individual Commercial, Group Commercial, or No MN APCD enrollment based on the primary medical insurance enrollment code from the MN APCD enrollment table. The MN APCD codes primary coverage based on the usual rules for "first dollar payer," so that individuals with multiple types of coverage have ties broken as: Commercial (any type), Medicare, Medicaid. Dual-eligible individuals were coded as enrolled in Medicare.

We coded individuals as having "No MN APCD enrollment" when there were no medical insurance records available for that month. This occurs when Onpoint was unable to link an enrollment record from any submitter, which can happen for several reasons. First, the individual may be uninsured, which is why we report No MN APCD Enrollment as an outcome. Alternatively, the individual could be covered by a non-reporting health plan such as the Indian Health Service, Veteran's Administration, Tricare, or health plans with under $3 million in claims for Minnesota residents. In rare cases, Onpoint is unable to link a person across different submitters, which "splits" an enrollee into two different member identifiers in the MN APCD, causing the existing identifier to appear to disenroll from Medicaid with no MN APCD enrollment. Death or relocation out of Minnesota can also cause an individual to have no MN APCD enrollment. Self-funded employer plans governed by the Employee Retirement and Security Act (ERISA) are also not statutorily required to submit data to the MN APCD but are

© 2023 Frenier C et al. *JAMA Network Open.*

encouraged to submit voluntarily. MDH estimates that the MN APCD data include about 40% of commercial lives during the study period.

No MN APCD enrollment suggests the possibility of uninsurance, but the data limitations above mean that we cannot definitively classify uninsurance in our data. To reduce potential errors from identifier splitting, death, and long-term relocation, we restricted the Figure sample to include only individuals who were observed coverage in at least one month between their exit date and September 2021, the latest available data in the MN APCD. We also note that the inability to observe all self-funded employer plans is a limitation of state-sponsored all payer claims databases generally and is not specific to the MN APCD.

© 2023 Frenier C et al. *JAMA Network Open.*
