# Supplementary material for: Insurance Coverage Transitions After Disenrollment From Medicaid in Minnesota
Source: JAMA Netw Open. 2023 Apr 21;6(4):e239379. doi: 10.1001/jamanetworkopen.2023.9379 (PMC10122164; doi:10.1001/jamanetworkopen.2023.9379)
Supplement: Supplement 2. — Data Sharing Statement [file jamanetwopen-e239379-s002.pdf]

Data Sharing Statement

Frenier. Insurance Coverage Transitions After Disenrollment From Medicaid in Minnesota. *JAMA Netw Open*. Published April 21, 2023. doi:10.1001/jamanetworkopen.2023.9379

Data
Data available: No

Additional Information
Explanation for why data not available: Data are owned by the Minnesota Department of Health. Contact health.apcd@state.mn.us for more information about the Minnesota All Payer Claims Database.
